# Mild versus moderate stages of Alzheimer's disease: three-year outcomes in a routine clinical setting of cholinesterase inhibitor therapy

**DOI:** 10.1186/s13195-016-0174-1

**Published:** 2016-02-17

**Authors:** Carina Wattmo, Lennart Minthon, Åsa K. Wallin

**Affiliations:** Clinical Memory Research Unit, Department of Clinical Sciences, Malmö, Lund University, SE-205 02 Malmö, Sweden

**Keywords:** Cognition, Activities of daily living, Cholinesterase inhibitors, Treatment effect, Alzheimer’s disease stages, Predictors, Longitudinal study, Statistical models

## Abstract

**Background:**

There is an increasing interest in cognitive and functional outcomes in the respective stages of Alzheimer’s disease (AD) and in novel therapies particularly for the milder phases of AD. Our aim was to describe and compare various aspects of disease progression in patients with mild versus moderate AD in routine clinical practice of cholinesterase inhibitor (ChEI) therapy.

**Methods:**

This 3-year, prospective, observational, multicentre study included 1021 participants. Of these, 734 had mild AD (Mini-Mental State Examination (MMSE) score, 20–26) and 287 had moderate AD (MMSE score, 10–19) at the start of ChEI treatment. At baseline and every 6 months, patients were assessed using cognitive, global, instrumental and basic activities of daily living (ADL) scales. Potential predictors of deterioration in moderate AD were analysed using mixed-effects models.

**Results:**

The change from baseline between participants with mild and moderate stages of AD after 3 years of ChEI therapy differed significantly on the Alzheimer’s Disease Assessment Scale-cognitive subscale (ADAS-cog) and basic ADL, but not using the MMSE and instrumental ADL scales. Protective independent factors for better cognitive long-term outcome in the group with moderate AD were older age, higher instrumental ADL ability, no antipsychotics, usage of non-steroidal anti-inflammatory drugs/acetylsalicylic acid, living with family member, lower education and a higher mean dose of ChEI. Apolipoprotein E genotype did not influence the rates of disease progression or the longitudinal outcomes. Prediction models were provided for moderate AD.

**Conclusions:**

More sensitive cognitive measures, such as the ADAS-cog scale, are required to detect a possibly faster deterioration among the participants with moderate AD. This study highlighted the clinical importance of instrumental ADL evaluations in patients at a mild stage of AD, and the importance of optimizing the ChEI dose even for individuals with moderate AD. Solitary living was a risk factor for faster cognitive decline, and probably expanded the need for formal care in the group with moderate AD. The patients with more advanced AD and presumably more pronounced neuroinflammation might have additional cognitive benefits from longer-term treatment with anti-inflammatory drugs.

## Background

The course of Alzheimer’s disease (AD) may be described in different stages because these patients exhibit different symptoms over time. However, the clinical presentation of AD and the progression of symptoms might show considerable heterogeneity among the affected individuals. In general, AD starts with mild impairments in memory, communication patterns and executive ability, and depression, which can be an early symptom of AD [1]. A marked reduction in the capacity to conduct more advanced instrumental activities of daily living (ADL) has also been observed in persons with mild AD [2]. Common symptoms in the moderate stage are disorientation of time and/or place, dyspraxia, dysgnosia and decreased judgement and skills with regard to basic ADL. Moreover, when the disease progresses, it is often accompanied by social withdrawal, changes in behaviour and psychotic symptoms [1].

Cholinesterase inhibitors (ChEIs) are the main recommended symptomatic treatment for patients with mild to moderate AD, and they are understood to work by increasing the level of acetylcholine in the neuronal synaptic clefts in the brain [3]. The level of short-term therapeutic response and longitudinal outcome may vary among those at the various stages of AD. Several studies have reported a better 6-month cognitive response to all three ChEIs for individuals in the moderate to moderate/severe stages [4–6], while long-term benefits of ChEI treatment were more pronounced in those patients with milder AD [7].

There is an increased interest in cognitive and functional outcomes according to the stages of AD, and in new therapies aimed at blocking the course of the disease especially in the early stages. The medical food Souvenaid (Nutricia N.V., Zoetermeer, The Netherlands) showed significant cognitive improvement compared with placebo in participants with mild AD exclusively, but failed in a cohort of patients with both mild and moderate AD [8]. Passive immunization with the anti-beta-amyloid antibody solanezumab yielded small but significant positive cognitive effects in individuals with mild AD [9]; however, the final phase 3 analyses observed no treatment–placebo differences in participants with either mild or moderate AD [10]. A phase 2 trial of idalopirdine + donepezil reported a significant improvement in cognitive ability in patients with moderate AD compared with donepezil therapy alone [11]. New longer studies are usually add-on studies – that is, performed using participants who are already being treated with ChEIs – because placebo-controlled trials lasting longer than 6 months in individuals with untreated AD are not permitted for ethical reasons. Knowledge of the longitudinal cognitive and functional outcomes in different stages of AD are of great importance for the evaluation of future add-on therapies (e.g. calculation of adequate sample sizes). New therapies that might modify AD progression require thorough assessment over several years; hence, the rates of change in well-designed observational studies can be used for comparisons.

Few earlier long-term studies focused on reporting data for ChEI-treated participants in the mild and moderate AD stages. Moreover, to our knowledge, no comparative studies of different aspects of disease progression in these stages over longer times have been reported. An observational study of the cognitive and ADL outcomes over 3 years of ChEI therapy of mild AD was described by our group [2]; the Alzheimer’s Disease Neuroimaging Initiative presented the 2-year cognitive decline in ChEI-treated patients in the mild stage of AD [12]; and an 18-month randomized controlled trial of tarenflurbil exclusively in participants with mild AD showed rates of change in cognition over time for the ChEI-treated and/or memantine-treated ‘placebo’ group [13]. No longitudinal studies have previously reported the progression in different domains or possible predictors that might affect the disease course in a cohort with exclusively moderate AD.

The present study aimed to compare various long-term outcomes between patients with mild or moderate AD in a routine clinical setting of ChEI therapy, and aimed to describe and predict cognitive, global and functional longitudinal progression in individuals with moderate AD. Disease progression regarding the group with mild AD was published previously [2].

## Methods

### Study and participants

The Swedish Alzheimer Treatment Study (SATS) is a 3-year, prospective, open, non-randomized multicentre study with the purpose of assessing long-term ChEI treatment (donepezil, rivastigmine and galantamine) in routine clinical practice. Several publications have previously reported various findings from the SATS [2, 6, 7, 14]. In total, 1258 participants with AD were recruited from 14 memory clinics in different areas of Sweden. Of these, 734 individuals were defined as having mild AD (Mini-Mental State Examination (MMSE) [15] score, 20–26) and 287 individuals as having moderate AD (MMSE score, 10–19) at the start of ChEI therapy (baseline) and were included in the present study.

The inclusion criteria were outpatients aged 40 years and older who received a clinical diagnosis of dementia as defined by the *Diagnostic and Statistical Manual of Mental Disorders*, 4th edition (DSM-IV) [16] and possible or probable AD according to the criteria of the National Institute of Neurological and Communicative Disorders and Stroke and the Alzheimer’s Disease and Related Disorders Association (NINCDS-ADRDA) [17]. In addition, the participants were required to be community dwelling with or without home-help services at the time of AD diagnosis, to have a responsible caregiver and to be capable of being evaluated using the MMSE scale at the initiation of ChEI treatment. Exclusion criteria were not fulfilling the diagnostic criteria for AD, already receiving active ChEI therapy or contraindications to ChEI. Concomitant medications other than ChEIs were recorded at baseline and allowed during the study, except for memantine. If memantine therapy was commenced, the individual dropped out from the SATS at that time point.

The SATS participants were investigated in a structured, follow-up programme over 3 years that investigated cognition, global performance and instrumental and basic ADL abilities, at the start of ChEI treatment, after 2 months (MMSE and global rating only) and every 6 months. Nurses trained to care for patients with dementia assessed the ADL capacity based on interviews with the caregiver. The dates of eventual nursing home placement and death were documented, as well as the date of, and reason for, any withdrawal from the SATS.

After inclusion in the study and the baseline evaluations, the participants were prescribed ChEI therapy as part of the ordinary Swedish health care system, in accordance with the approved product labelling. The SATS is an observational study and the choice of ChEI agent and dose was left entirely up to the dementia specialist’s discretion and professional judgement. The ChEI dose was recorded after 2 months of treatment and then semi-annually after baseline. If the patient stopped taking the ChEI, the individual was excluded from the study at that time point.

### Ethics, consent and permissions

All patients and/or their caregivers gave their written informed consent to participate in the SATS, which was conducted according to the provisions of the Helsinki Declaration and was approved by the Ethics Committee of Lund University, Sweden.

### Outcome measures

Cognitive status was assessed using the MMSE scale (with scores ranging from 0 to 30; a lower score indicates more impaired cognition) and the Alzheimer’s Disease Assessment Scale—cognitive subscale (ADAS-cog) [18] (0–70 points; a lower score indicates higher cognitive ability). The Clinician Interview-Based Impression of Change (CIBIC) [19] was used as a global rating of ‘change from the start of ChEI treatment’. The evaluations were performed at all intervals using a 7-point scale that varied from 1 (very much improved) to 7 (marked worsening). Three groups of response were defined at each CIBIC interval: 1–3 indicated improvement, 4 indicated no change and 5–7 indicated worsening. No guidelines or descriptors were provided to define the individual ratings. The classification between, for example, minimally improved or very much improved was left to the dementia specialist’s clinical judgement.

The functional capacity was assessed using the Instrumental Activities of Daily Living (IADL) scale [20], which comprises eight items: ability to use the telephone, shopping, food preparation, housekeeping, ability to do laundry, mode of transportation, responsibility for own medications and ability to handle finances. Each item was scored from 1 (no impairment) to 3–5 (severe impairment), thus allowing a total range of 8–31 points. Basic ADL was measured by the Physical Self-Maintenance Scale (PSMS) [20] comprising six items: toilet, feeding, dressing, grooming, physical ambulation and bathing. Each item was scored from 1 (no impairment) to 5 (severe impairment), thus allowing a total range of 6–30 points.

For each follow-up visit, we calculated the mean MMSE, ADAS-cog, IADL and PSMS changes from baseline with 95 % confidence intervals (CI). To facilitate comparisons of these rates, we converted the change in score to positive values (indicating improvement) and negative values (indicating worsening). The proportions of improved/unchanged SATS patients, predefined as those who demonstrated an improvement or no change (≥0 points difference) at the respective evaluation, were also calculated for the MMSE, ADAS-cog, IADL and PSMS scales.

Nursing home placement was defined as the permanent admission to a licensed skilled nursing facility with 24-hour care; that is, rehabilitative or respite care was not included. If hospitalization occurred before nursing home entry, the date of hospital admission was used. Using the 12-digit personal identity number assigned to each resident of Sweden, we determined whether each participant in the study was still alive on 31 December 2013 with the help of the Swedish population register (Swedish Tax Agency). If not, the date of death was recorded.

### Statistical analyses

The IBM Statistical Package for the Social Sciences (SPSS) for Windows (version 22.0; IBM Corporation, Armonk, NY, USA) was used to perform the statistical analyses. The level of significance was defined as *p* <0.05, unless otherwise specified, and all tests were two-tailed. Observed-case analyses were used to avoid overestimation of the therapeutic effect by imputing earlier, better outcome scores in a long-term study of a progressively deteriorating disease. Parametric tests were used because of the large sample size and the approximately normally distributed continuous potential predictors. Independent-sample *t* tests were used to compare the differences between the means for two groups, and chi-square tests were conducted to analyse categorical variables. Pearson’s correlation coefficient was calculated to investigate any linear associations between continuous variables.

Mixed, linear and non-linear fixed and random coefficient regression models using the subject as a hierarchical variable (to consider the intra-individual correlation) were performed. In addition, the mixed-effects models took into account the varying number of assessments available for each patient and unequal time intervals between follow-ups, which are the usual concerns in longitudinal studies. The individuals who discontinued the study contributed information during their time of participation; hence, we considered the trajectories of all patients in the SATS.

Time was defined as the exact number of months between the start of ChEI therapy and each visit, which implies that all data points were used at the actual time intervals. To adjust for baseline differences, the initial cognitive, instrumental or basic ADL scores for each individual and their interaction with linear and quadratic terms for time in the study (to enable a non-linear rate of change in the models) were included as fixed effects; that is, time in months (and time in months^2^) × MMSE (ADAS-cog, IADL or PSMS) baseline score. Thus, the dependent variables were the cognitive or functional scores assigned at the second and subsequent evaluations for each participant; the mixed-effects models do not intend to predict the scores at the initiation of ChEI treatment. The random terms were an intercept and time in months, with a variance components covariance matrix. Several potential socio-demographic and clinical predictors were included as fixed effects in the models, such as sex, age at the start of ChEI therapy, clinician’s estimate of age at AD onset, years of education, presence of the apolipoprotein E (APOE) ε4 allele (no/yes), solitary living (no/yes), number of medications at baseline, and specific concomitant medications (no/yes for each group) including antihypertensive/cardiac therapy, antidiabetics, asthma medication, thyroid therapy, lipid-lowering agents, oestrogens, non-steroidal anti-inflammatory drugs (NSAIDs)/acetylsalicylic acid, antidepressants, antipsychotics and anxiolytics/sedatives/hypnotics. The effect of ChEIs was analysed using the drug agents (coded as a set of dummy variables) and dosages. The ChEI dose could vary during the treatment period for an individual patient and between patients; therefore, the mean dose used during the entire follow-up period was calculated for each participant. In cases of drop-out, the mean dose used during the individual’s time of participation in the SATS was calculated. To obtain a similar metric for percentage maximum dosage for each of the three ChEIs, the mean dose was divided by the maximum recommended dose for each drug; that is, 10 mg for donepezil, 12 mg for rivastigmine (oral administration) and 24 mg for galantamine. The term ‘ChEI agent × dose’ was also included in the models. Furthermore, some potential interactions (gender, age or education) with disease severity at baseline or with time in the study were included in the models. Non-significant variables (*p* >0.05) were eliminated in a backward stepwise manner. The hierarchical principle was applied in the mixed-effects models; variables that appeared in significant interactions were not considered for elimination.

## Results

### Socio-demographic and clinical characteristics according to stage of AD

The 1021 SATS participants were divided into two groups according to their cognitive status at the start of ChEI therapy (baseline): mild AD (MMSE score, 20–26; *n* = 734 (72 %)) and moderate AD (MMSE score, 10–19; *n* = 287 (28 %)). The socio-demographic and clinical characteristics of the two groups are presented in Table 1. In the mild cohort, the proportion of antipsychotic medications was lower (χ^2^(1) = 6.69; *p* = 0.013). The patients with mild AD also had significantly more years of education, on average, compared with those in the moderate group (*t*_(1017)_ = 3.82; *p* <0.001).

**Table 1:** Socio-demographic and clinical characteristics (*n* = 1021)

Variable	Mild AD (*n* = 734, 72 %)	Moderate AD (*n* = 287, 28 %)	*p*
Female sex	473/64 %	181/63 %	0.717
APOE ε4 carrier, (*n* = 999)	493/69 %	186/66 %	0.452
Solitary living at baseline	267/36 %	88/31 %	0.093
Completion rate after 3 years	306/42 %	78/27 %	<0.001
Antihypertensives/cardiac therapy	290/40 %	124/43 %	0.288
Antidiabetics	38/5 %	12/4 %	0.629
Asthma medication	28/4 %	16/6 %	0.231
Thyroid therapy	65/9 %	20/7 %	0.378
Lipid-lowering agents	94/13 %	24/8 %	0.050
Oestrogens	52/7 %	17/6 %	0.580
NSAIDs/acetylsalicylic acid	221/30 %	84/29 %	0.820
Antidepressants	183/25 %	74/26 %	0.810
Antipsychotics	26/4 %	21/7 %	0.013
Anxiolytics/sedatives/hypnotics	111/15 %	37/13 %	0.429
Estimated age at onset (years)	72.3 ± 7.1	72.0 ± 7.8	0.544
Estimated duration of AD at baseline (years)	2.9 ± 2.0	3.4 ± 2.3	0.005
Age at first assessment (years)	75.2 ± 6.8	75.3 ± 7.4	0.788
Education (years)	9.6 ± 2.6	9.0 ± 2.2	<0.001
MMSE score at baseline	23.4 ± 2.0	16.4 ± 2.2	<0.001
ADAS-cog score (0–70) at baseline	17.5 ± 6.7	29.3 ± 8.3	<0.001
IADL score at baseline	14.7 ± 5.0	19.1 ± 5.1	<0.001
PSMS score at baseline	7.2 ± 1.9	8.3 ± 2.9	<0.001
Number of concomitant medications at baseline	2.9 ± 2.4	3.0 ± 2.6	0.604
Length in the SATS (months)	24.2 ± 12.9	20.4 ± 13.0	<0.001
Mean dose of ChEI during the follow-up period (mg)			
Donepezil (*n* = 518)^a^	6.9 ± 1.7 (48 %)	6.9 ± 1.8 (57 %)	0.761
Rivastigmine (*n* = 212)^a^	6.2 ± 2.1 (22 %)	6.0 ± 2.2 (17.5 %)	0.536
Galantamine (*n* = 291)^a^	15.3 ± 3.6 (30 %)	14.7 ± 4.0 (25.5 %)	0.198

Data presented as *n*/% or mean ± standard deviation

^a^Percentage of patients in each group that received the specific ChEI agent in parentheses (chi-square test; *p* = 0.035)

*AD* Alzheimer’s disease, *ADAS-cog* Alzheimer’s Disease Assessment Scale—cognitive subscale, *APOE* apolipoprotein E, *ChEI* cholinesterase inhibitor, *IADL* Instrumental Activities of Daily Living scale, *MMSE* Mini-Mental State Examination, *NSAID* non-steroidal anti-inflammatory drug, *PSMS* Physical Self-Maintenance Scale, *SATS* Swedish Alzheimer Treatment Study

The 3-year completion rate was higher (χ^2^(1) = 15.98; *p <*0.001) and the mean time of participation in the study was longer (*t*_(1019)_ = 4.24; *p* <0.001) for the cohort with mild AD compared with moderate AD. A higher percentage of individuals with moderate AD received donepezil and a lower percentage rivastigmine compared with those in the group with mild AD (χ^2^(2) = 6.70; *p* = 0.035). No differences in sex, APOE genotype, age at onset or at baseline, ChEI dose or use of other medications at baseline were detected between the two groups.

### Comparisons of longitudinal outcomes between mild and moderate AD

**Fig. 1:**
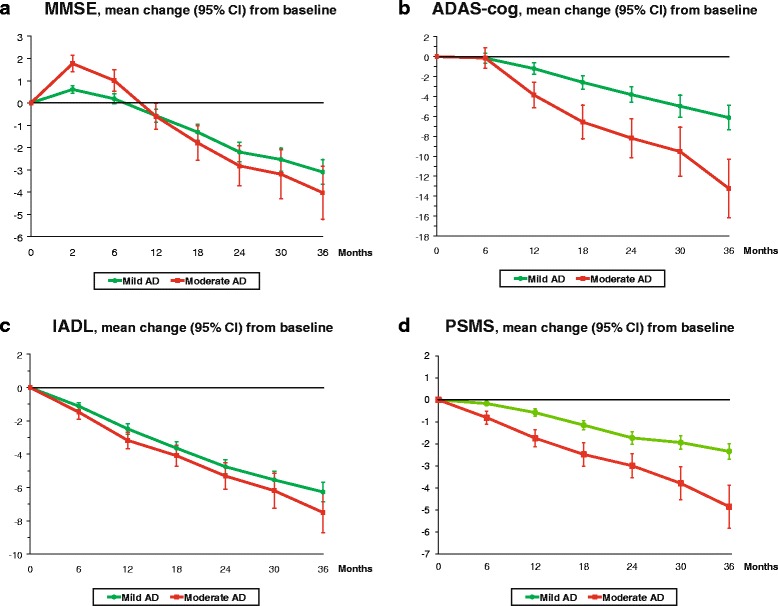
Cognitive and functional outcomes over 3 years of ChEI treatment. **a** Mean changes in MMSE score with 95 % CI from the start of ChEI therapy over 3 years according to the stage of AD. The SATS patients with moderate AD exhibited a better short-term cognitive outcome after 2 months (*p* <0.001) and 6 months (*p* = 0.003) of therapy. No significant difference was found between the two disease stages at the other evaluations. **b** Mean changes in ADAS-cog score with 95 % CI from the start of ChEI therapy over 3 years according to the stage of AD. The patients with mild AD showed a more positive longitudinal cognitive outcome from the 12-month assessment (*p* <0.001). **c** Mean changes in IADL score with 95 % CI from the start of ChEI therapy over 3 years according to the stage of AD. The patients with mild AD exhibited a better functional outcome after 12 months (*p* = 0.021). No significant difference was detected between the two disease stages at the other evaluations. **d** Mean changes in PSMS score with 95 % CI from the start of ChEI therapy over 3 years according to the stage of AD. The patients with mild AD showed a more favourable long-term outcome in basic ADL from the 6-month assessment (*p* <0.001). *AD* Alzheimer’s disease, *ADAS-cog* Alzheimer’s Disease Assessment Scale—cognitive subscale, *CI* confidence interval, *IADL* Instrumental Activities of Daily Living scale, *MMSE* Mini-Mental State Examination, *PSMS* Physical Self-Maintenance Scale

**Fig. 2:**
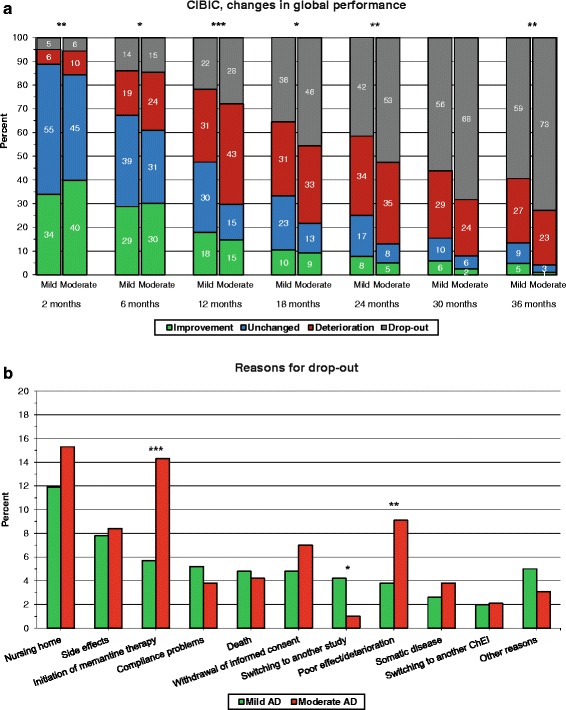
Proportion of SATS participants. **a** Proportion of patients according to differences in treatment response in global performance (CIBIC) from the start of ChEI therapy over 3 years for mild vs. moderate AD (***p <0.001, **0.001 ≤ *p* <0.01, *p <0.05). CIBIC score 1–3 was considered as improvement, 4 as unchanged and 5–7 as deterioration. **b** Proportion of patients who discontinued the study for various reasons according to the stage of their AD. Initiation of memantine therapy (*p <*0.001) and poor effect/deterioration (*p =* 0.002) were more frequent reasons for drop-out in the cohort with moderate AD; switching to another study (*p =* 0.010) was more common among the patients with mild AD. No significant difference between the disease stages was observed for the other reasons for drop-out. *AD* Alzheimer’s disease, *ChEI* cholinesterase inhibitor, *CIBIC* Clinician Interview-Based Impression of Change

The mean MMSE, ADAS-cog, IADL and PSMS changes from baseline scores during 3 years for the SATS patients with mild and moderate AD are shown in Fig. 1a–d. After 3 years of ChEI treatment, the mean decline (95 % CI) from baseline for mild and moderate AD was: MMSE score, 3.1 (2.5–3.7) vs. 4.0 (2.8–5.2) points (*t*_(368)_ = 1.45; *p* = 0.148); ADAS-cog score, 6.1 (4.9–7.4) vs. 13.2 (10.3–16.2) points (*t*_(329)_ = 4.84; *p* <0.001); IADL score, 6.3 (5.7–6.9) vs. 7.5 (6.3–8.7) points (*t*_(354)_ = 1.87; *p* = 0.063); and PSMS score, 2.3 (2.0–2.7) vs. 4.9 (3.9–5.8) points (*t*_(355)_ = 4.82; *p* <0.001). The changes in global performance (CIBIC) from the initiation of ChEIs and over the 3-year study are shown in Fig. 2a. After 1 year of therapy, global improvement or no change was observed in 61 % of the remaining mild cohort vs. 41 % of the remaining moderate cohort (χ^2^(1) = 23.75; *p <*0.001); after 2 years, 43 % vs. 27 % (χ^2^(1) = 10.37; *p =* 0.001); and after 3 years, 33 % vs. 15 % of the remaining individuals were improved/unchanged (χ^2^(1) = 9.15; *p =* 0.002). No significant linear associations were found between cognitive, IADL or basic ADL abilities at the baseline, or their rates of change during the study, and the mean dose of ChEI.

The participants with mild and moderate AD, respectively, were further divided into APOE genotypes. No significant difference in ADAS-cog or PSMS change after 3 years of ChEI treatment was observed between the mild non-carriers and carriers of the ε4 allele, or between the moderate non-carriers and carriers of the ε4 allele. No difference in change from baseline after 3 years was found in MMSE and IADL scores between the four groups. The proportion of globally improved/unchanged patients was not different between non-carriers and carriers of the ε4 allele either with mild AD or moderate AD.

### End-points in mild vs. moderate AD

Overall, 428 participants (58 %) with mild AD and 209 participants (73 %) with moderate AD did not complete the 3-year study (χ^2^(1) = 15.98; *p <*0.001). The reasons for drop-out are shown in Fig. 2b. Initiation of memantine therapy (χ^2^(1) = 20.26; *p <*0.001) and poor effect/deterioration (χ^2^(1) = 11.33; *p =* 0.002) were more frequent reasons in the cohort with moderate AD, while switching to another study (χ^2^(1) = 6.47; *p =* 0.010) was more common among patients with mild AD. Thirty-eight (5 %) of the individuals with mild AD and 11 individuals (4 %) with moderate AD dropped out of the study because of compliance problems (χ^2^(1) = 0.82; *p* = 0.419).

**Fig. 3:**
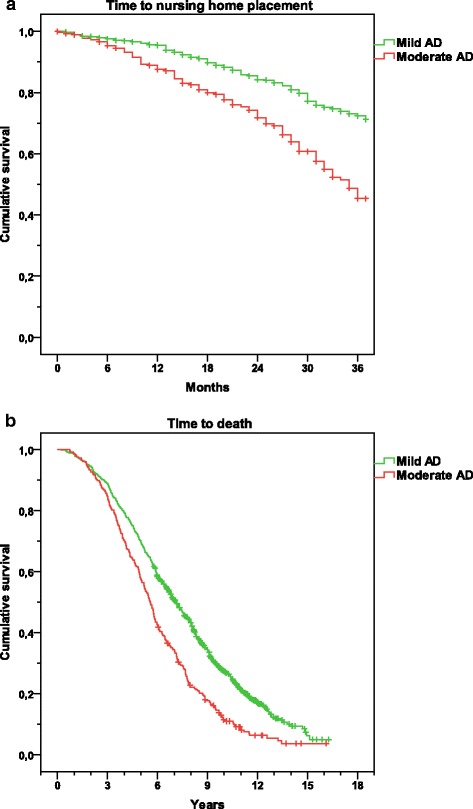
Time to end-points. **a** Kaplan–Meier graph for the distribution of time from the start of ChEI therapy (approximately time of AD diagnosis) to nursing home placement for the SATS group with mild vs. moderate AD. A log–rank test found a longer time to institutionalization for patients with mild AD (*p* <0.001). **b** Kaplan–Meier graph for the distribution of time from the start of ChEI therapy to death according to stage of AD. A log-rank test showed a shorter life expectancy for the patients with moderate AD (*p* <0.001). *AD* Alzheimer’s disease

During the SATS, 141 participants (19 %) with mild AD and 91 participants (32 %) with moderate AD (χ^2^(1) = 18.35; *p <*0.001) were admitted to nursing homes. Figure 3a presents the Kaplan–Meier graph for the distribution of time from baseline to nursing home placement for the mild and moderate groups (log–rank test, *p* <0.001). However, the mean time (95 % CI) from the start of ChEI treatment to institutionalization was similar for patients with mild and moderate AD (20.0 (18.4–21.6) months vs. 19.0 (16.8–21.1) months; *p* = 0.449).

After the 3-year study, 88 individuals (12 %) with mild AD and 47 individuals (16 %) with moderate AD had died (*p* = 0.065), and after up to 16 years of follow-up 578 individuals (79 %) and 263 individuals (92 %) (χ^2^(1) = 23.61; *p <*0.001), respectively, had died. Figure 3b presents the Kaplan–Meier graph for the distribution of time from the initiation of ChEI therapy to death for patients with mild and moderate AD (log–rank test, *p* <0.001). The mean survival time from the baseline was 6.2 (5.9–6.4) years and 5.5 (5.2–5.8) years (*t*_(839)_ = 3.34; *p* = 0.001) for individuals with mild and moderate AD, respectively.

### Longitudinal outcomes and prediction models in moderate AD

The mean MMSE, ADAS-cog, IADL and PSMS actual scores and the changes from baseline scores during 3 years are presented in Table 2. The proportions of participants with moderate AD who exhibited improvement or remained unchanged at each visit according to these measures are also reported. The aforementioned outcomes for the patients with mild AD in the SATS have been published previously [2].

**Table 2:** Changes in cognitive and functional abilities during 3 years of ChEI therapy in patients with moderate AD

Variable	2 months (*n* = 273)	6 months (*n* = 250)	12 months (*n* = 214)	18 months (*n* = 169)	24 months (*n* = 140)	30 months (*n* = 96)	36 months (*n* = 78)
Completion rate (%)	95.1	87.1	74.6	58.9	48.8	33.4	27.2
MMSE score^a^	18.2 (17.7, 18.6)	17.4 (16.8, 18.0)	15.8 (15.1, 16.4)	14.7 (13.8, 15.5)	13.9 (12.9, 14.9)	13.7 (12.5, 14.9)	12.8 (11.5, 14.1)
ADAS-cog score (0–70)^a^		28.8 (27.5, 30.2)	32.3 (30.6, 34.0)	35.0 (32.8, 37.1)	35.9 (33.3, 38.5)	36.4 (33.2, 39.5)	39.4 (35.7, 43.1)
IADL score^a^		20.6 (20.0, 21.2)	22.3 (21.6, 22.9)	23.4 (22.6, 24.1)	24.3 (23.6, 25.0)	25.1 (24.3, 25.8)	26.2 (25.5, 27.0)
PSMS score^a^		9.1 (8.6, 9.5)	9.8 (9.3, 10.3)	10.5 (9.9, 11.2)	10.8 (10.2, 11.4)	11.6 (10.8, 12.4)	12.6 (11.6, 13.6)
MMSE score, change from baseline^a^	1.77 (1.40, 2.14)	1.01 (0.52, 1.50)	−0.60 (−1.18, −0.02)	−1.79 (−2.58, −1.01)	−2.82 (−3.73, −1.92)	−3.20 (−4.29, −2.11)	−4.03 (−5.22, −2.83)
ADAS-cog score (0–70), change from baseline^a^		−0.14 (−1.19, 0.90)	−3.85 (−5.13, −2.57)	−6.56 (−8.26, −4.86)	−8.18 (−10.14, −6.23)	−9.52 (−11.98, −7.06)	−13.22 (−16.16, −10.28)
IADL score, change from baseline^a^		−1.48 (−1.89, −1.07)	−3.17 (−3.66, −2.67)	−4.09 (−4.73, −3.46)	−5.31 (−6.09, −4.52)	−6.19 (−7.23, −5.15)	−7.50 (−8.71, −6.30)
PSMS score, change from baseline^a^		−0.81 (−1.11, −0.51)	−1.74 (−2.13, −1.36)	−2.48 (−3.02, −1.94)	−2.99 (−3.53, −2.45)	−3.79 (−4.55, −3.04)	−4.85 (−5.83, −3.88)
MMSE score, improved/unchanged patients (%)	74.5	66.4	51.5	45.2	32.8	36.7	28.9
ADAS-cog score (0–70), improved/unchanged patients (%)		53.4	37.6	29.9	23.6	16.9	10.9
IADL score, improved/unchanged patients (%)		46.0	23.3	17.2	14.7	9.2	8.1
PSMS score, improved/unchanged patients (%)		62.4	45.5	34.9	23.3	21.8	17.6

For clarity, clinical improvements for all scales have been tabulated as positive changes from the start of ChEI therapy (baseline)

^a^Mean (95 % confidence interval)

*AD* Alzheimer’s disease, *ADAS-cog* Alzheimer’s Disease Assessment Scale-cognitive subscale, *ChEI* cholinesterase inhibitor, *IADL* Instrumental Activities of Daily Living scale, *MMSE* Mini-Mental State Examination, *PSMS* Physical Self-Maintenance Scale

To enable analyses of a non-linear rate of cognitive or functional change in the mixed-effects models, only individuals with three or more assessments (*n* = 249, 86.8 %) were included. The models were studied (1129 data points) to identify the socio-demographic and clinical variables that affected the long-term trajectories of patients with moderate AD. The percentages of variance that accounted for the dependent variable, regarding all fixed predictors, were 38.5 % for MMSE, 47.8 % for ADAS-cog, 56.0 % for IADL and 39.9 % for PSMS, indicating a good fit of the models (*p* <0.001). The mixed-effects models, significant predictors and unstandardized β coefficients with 95 % CI are presented in Tables 3 and 4. Older participants and those with more preserved IADL capacity at baseline exhibited a more favourable longitudinal cognitive outcome. A lower level of education and a higher mean dose of ChEI during the study (irrespective of drug agent) were independent predictors of better cognitive ability as assessed by the ADAS-cog score in individuals with moderate AD. In the mixed-effects models, living with a family member implied a mean reduction of almost 3 points on ADAS-cog, and the presence of NSAIDs/acetylsalicylic acid therapy implied a decrease of more than 2 points on the ADAS-cog outcome. Usage of antipsychotics estimated an average score 2 points lower as measured by the MMSE scale. The interaction effects of these variables with time were not significant; that is, no additional increase in cognitive impairment associated with the aforementioned variables was detected during the 3-year study. Less functional progression was related to better cognitive performance at baseline and a higher mean ChEI dose.

**Table 3:** Factors affecting the long-term outcome with MMSE or ADAS-cog score as dependent variables

	MMSE			ADAS-cog		
Significant predictors in final mixed models^a^	β	95 % CI	*p*	β	95 % CI	*p*
Percentage of variance accounted for, all fixed terms	38.5 %, *p* <0.001			47.8 %, *p* <0.001		
Fixed terms						
Intercept	−0.936	−6.009, 4.137	0.717	21.058	8.622, 33.493	0.001
Time in months from baseline	−0.887	−1.264, −0.509	<0.001	−0.215	−0.651, 0.221	0.333
Baseline assessment score	0.937	0.760, 1.114	<0.001	0.636	0.508, 0.765	<0.001
Time in months × baseline assessment score	0.016	0.002, 0.030	0.030	0.015	0.005, 0.025	0.003
Background variables						
Solitary living (no = 0, yes = 1)			ns	2.882	0.887, 4.877	0.005
NSAIDs/acetylsalicylic acid (no = 0, yes = 1)			ns	−2.368	−4.353, −0.383	0.020
Antipsychotics (no = 0, yes = 1)	−1.985	−3.346, −0.625	0.004			ns
Education (years)			ns	−0.374	−0.865, 0.118	0.136
Time in months × education (years)			ns	0.048	0.008, 0.089	0.020
Age at first assessment (years)	0.057	0.006, 0.109	0.030	−0.160	−0.288, −0.033	0.014
Time in months × age	0.005	0.001, 0.009	0.016			ns
IADL score at baseline	−0.078	−0.156, −0.001	0.048	0.241	0.043, 0.439	0.017
ChEI dose^b^			ns	−0.057	−0.105, −0.010	0.018
Random terms (variance)						
Intercept	4.671	3.445, 6.334	<0.001	11.382	5.851, 22.143	0.003
Time in months	0.029	0.021, 0.040	<0.001	0.174	0.128, 0.237	<0.001

Sex, apolipoprotein E genotype, age at onset, Physical Self-Maintenance Scale score at baseline, number of medications and the other specific concomitant medications used at baseline, as well as the variable comparing the ChEI agents, were not significant predictors in the models. β values were unstandardized and are expressed per 1 unit increase for continuous variables and for the condition present in dichotomous variables

^a^Baseline assessment score = MMSE or ADAS-cog, respectively

^b^Mean percentage of the maximum recommended dose; that is, 10 mg for donepezil, 12 mg for rivastigmine, and 24 mg for galantamine

*ADAS-cog* Alzheimer’s Disease Assessment Scale—cognitive subscale, *ChEI* cholinesterase inhibitors, *CI* confidence interval, *IADL* Instrumental Activities of Daily Living, *MMSE* Mini-Mental State Examination, *ns* not significant, *NSAID* non-steroidal anti-inflammatory drug

**Table 4:** Factors affecting the long-term outcome with IADL or PSMS score as dependent variables

	IADL			PSMS		
Significant predictors in final mixed models^a^	β	95 % CI	*p*	β	95 % CI	*p*
Percentage of variance accounted for, all fixed terms	56.0 %, *p* <0.001			39.9 %, *p* <0.001		
Fixed terms						
Intercept	2.375	−3.349, 8.099	0.414	4.844	2.154, 7.534	<0.001
Time in months from baseline	0.515	0.407, 0.623	<0.001	0.188	0.162, 0.213	<0.001
Baseline assessment score	1.431	0.926, 1.936	<0.001	0.900	0.789, 1.011	<0.001
Baseline assessment score^2^	−0.016	−0.029, −0.003	0.019			ns
Time in months × baseline assessment score	−0.013	−0.017, −0.008	<0.001			ns
Time in months^2^ × baseline assessment score	−0.0001	−0.0002, −0.00002	0.010			ns
Background variables						
MMSE score at baseline	−0.195	−0.374, −0.016	0.033	−0.203	−0.339, −0.067	0.004
ChEI dose^b^	−0.025	−0.048, −0.002	0.037	−0.019	−0.035, −0.003	0.021
Random terms (variance)						
Intercept	5.089	3.832, 6.757	<0.001	1.762	1.048, 2.963	<0.001
Time in months	0.008	0.005, 0.012	<0.001	0.017	0.013, 0.024	<0.001

Sex, apolipoprotein E genotype, solitary living, age at onset, age at baseline, years of education, number of medications and specific concomitant medications used at baseline, as well as the variable comparing the ChEI agents, were not significant predictors in the models. β values were unstandardized and are expressed per 1 unit increase for continuous variables and for the condition present in dichotomous variables

^a^Baseline assessment score = IADL or PSMS, respectively

^b^Mean percentage of the maximum recommended dose; that is, 10 mg for donepezil, 12 mg for rivastigmine and 24 mg for galantamine

*ChEI* cholinesterase inhibitors, *CI* confidence interval, *IADL* Instrumental Activities of Daily Living, *MMSE* Mini-Mental State Examination, *ns* not significant, *PSMS* Physical Self-Maintenance Scale

Non-linear regression models for calculation of the predicted MMSE, ADAS-cog, IADL or PSMS score for a group of ChEI-treated patients with moderate AD, based on the respective baseline score, are provided. These equations are intended to predict the scores at subsequent evaluations over a 3-year period. The models explained a substantial degree of variance in the data set – that is, they demonstrated a good fit: MMSE, *R*^2^ = 0.341, *R* = 0.584, *p* <0.001; ADAS-cog, *R*^2^ = 0.466, *R* = 0.682, *p* <0.001; IADL, *R*^2^ = 0.562, *R* = 0.750, *p* <0.001; and PSMS, *R*^2^ = 0.394, *R* = 0.628, *p* <0.001. These non-linear regression model equations are as follows:

Predicted MMSE score,

$$ \widehat{Y}=14.9503-\left(0.2806\times t\right)-\left(0.7801\times {x}_i\right)+\left(0.0027\times {t}^2\right)+\left(0.0613\times {x_i}^2\right) $$

Predicted ADAS-cog score,

$$ \widehat{Y}=5.4994+\left(0.1014\times t\right)+\left(0.7152\times {x}_i\right)+\left(0.0120\times t{x}_i\right) $$

Predicted IADL score,

$$ \widehat{Y}=1.8953+\left(0.5558\times t\right)+\left(0.9243\times {x}_i\right)-\left(0.0191\times t{x}_i\right) $$

Predicted PSMS score,

$$ \widehat{Y}=-0.2714+\left(0.2239\times t\right)+\left(1.0363\times {x}_i\right)-\left(0.0121\times t{x}_i\right) $$

where *t* is the time in months between the baseline score and the actual visit, and *x*_*i*_ is the baseline MMSE (ADAS-cog, IADL or PSMS) score.

## Discussion

In this study conducted in routine clinical practice, varying 3-year outcomes between participants in the two different stages of AD were demonstrated depending on the measures used. No significant difference was found between the MMSE and IADL scores, whereas the deterioration using ADAS-cog and basic ADL scales was faster in the group with moderate AD. Nursing home placement during the study was less frequent for the patients with mild AD, but the proportion of deceased individuals between the two stages did not differ after 3 years. Using mixed-effects models, risk factors for worse cognitive long-term outcome in moderate AD were antipsychotic medications, no usage of NSAIDs/acetylsalicylic acid, living alone, younger age, more years of education and lower IADL capacity. A higher mean ChEI dose was associated with slower cognitive and functional decline. Prediction models with the variance explained to a larger extent were also presented for the moderate cohort.

There has been increased interest in disease progression and response to therapies in various stages of AD after the solanezumab phase 2 [9] and Souvenaid [8] trials that reported small, but significant, positive outcomes for participants with mild AD. The solanezumab phase 3 trials [10] showed no placebo-treatment differences in cognition; however, the ‘placebo’ (the majority of patients were treated with ChEIs and/or memantine) group with mild AD deteriorated, on average, 5.1 points on the ADAS-cog scale and 2.4 points in MMSE score after 18 months. The corresponding decline in the SATS was 2.6 points and 1.3 points [2]. The mean deterioration for the moderate ‘placebo’ group in the 18-month solanezumab trial was 10.9 points on the ADAS-cog scale and 5.8 points in MMSE score, whereas the declines in SATS participants were 6.6 points and 1.8 points, respectively. A 6-month randomized controlled trial of idalopirdine in patients with moderate AD found a mean deterioration of 1.38 points on the ADAS-cog scale in the donepezil-treated ‘placebo’ group [11], while the current study demonstrated an average deterioration of 0.14 points after 6 months. The participants in both the solanezumab and idalopirdine trials were on stable treatment with ChEIs and/or memantine before their inclusion in the trials [10, 11]. The slower rate of cognitive impairment over time reported in the SATS might reflect a positive response to continuous ChEI therapy in the first months after initiation; therefore, it may be important to consider the point when treatment with ChEIs was started when comparing measures of disease progression between studies.

The cognitive rates of change over time between the patients with mild vs. moderate AD in the present study differed appreciably, depending on the chosen test instrument—MMSE or ADAS-cog, which are commonly used during these stages of AD. The selected assessment scale can affect the detected profile and rate of deterioration [21]. For example, a ceiling effect (the scale is less sensitive in the detection of actual changes during the very mild stage) and a floor effect (inability to evaluate severely impaired persons adequately) might affect the apparent trajectories. Moreover, a change in the score on a certain test is expected to be greater at the level of function, at which the scale measures the individual’s capacities most accurately [22]. Our findings indicate that the items in the more complex ADAS-cog scale are better adapted to measure the cognitive performance of the cohort with moderate AD, and that the MMSE seems to be less sensitive in describing the rate of change in cognition for participants with mild or moderate AD. This calls for the use of other scales that are more sensitive to changes in the progression of the disease (e.g. ADAS-cog). In clinical practice, the MMSE test is simpler to administer than the ADAS-cog; however, the latter is better when it comes to measuring change from a scientific point of view. The higher percentage of the explained variance observed for the ADAS-cog score compared with MMSE score (48 % vs. 38 %) in our mixed-effects models strengthens this observation. Knowledge of the expected long-term progression rate in various scales and stages of AD is clinically important for patient prognosis and anticipated care needs (i.e. whether the individual is declining at a rapid or slow rate) and for the assessment of the effectiveness of new therapies (e.g. calculation of sample sizes).

Surprisingly, no significant difference in the rate of IADL deterioration was detected between participants with mild and moderate AD after 3 years in this study. Our previous study of mild AD found that 45–65 % of the patients needed assistance with IADL tasks at baseline [2]. The current study showed that nursing home placement was less frequent among individuals with mild AD. However, the time to institutionalization for those who were admitted and the proportion of deaths over the 3-year study did not differ between the disease stages. A recent review reported that IADL impairment occurred early at a stage of mild cognitive impairment [23]. These observations stress the need for functional evaluations during the early phases of cognitive decline. Information about the patient’s IADL abilities is important knowledge for family members, clinicians and community-based services, for example, to assess safety issues (driving, management of own finances and medication intake) and to provide an adequate amount of formal care to postpone nursing home placement.

The significant predictors of progression in the moderate stage of AD varied somewhat between the MMSE and ADAS-cog scales in the present study. A higher level of education significantly precipitated the cognitive impairment measured by the ADAS-cog scale, but not the MMSE score, over time. Individuals with more years of education are expected to have a higher premorbid cognitive status. Hence, they might have a relatively larger burden of AD pathology and a more advanced level of the disease when dementia is clinically manifest [24]. This is one explanation for the influence of education level on the more demanding ADAS-cog scale in our group of participants with moderate AD. More years of education in patients with mild or mild-to-moderate AD have been related to faster cognitive deterioration using both MMSE and ADAS-cog scales [2, 7, 25], indicating a greater neurodegeneration at the time of AD diagnosis and delayed initiation of anti-dementia therapy compared with those with a lower level of education.

In the current analyses of moderate AD, the presence of NSAIDs/acetylsalicylic acid medications was a protective factor for a better longitudinal outcome in cognition (ADAS-cog score). We also found this protective factor in our entire SATS cohort with mild to moderate AD [7], but not in participants exclusively in the mild stage of AD [2]. Neuroinflammation has been suggested to participate in the pathogenetic cascades of AD, and a recent study from our group demonstrated that cerebral inflammation was an independent predictor of earlier death in AD [26]. The findings lead to the hypothesis that anti-inflammatory drugs could act as possible preventive or therapeutic approaches. A recent meta-analysis of observational studies shows that use of NSAIDs and aspirin, particularly over longer periods, could significantly prevent the occurrence of AD [27]. However, a review of randomized trials found no effect of NSAID therapy on cognitive decline compared with placebo in patients with mild to moderate AD; however, none of the included studies was longer than 12 months [28]. Nor was any beneficial effect observed in an 18-month trial of tarenflurbil in mild AD exclusively [13]. An explanation for these negative findings is that the follow-up period was not sufficiently long enough for a therapeutic effect to emerge in comparison with the 3-year perspective of the SATS. In addition, individuals with more advanced disease and neuroinflammation might have additional benefit from longer-term treatment with anti-inflammatory drugs and their potential for slowing AD progression.

In this study, the use of antipsychotics and solitary living were found to be independent risk factors for a more rapid cognitive deterioration in moderate AD; however, these predictors were not found to be significant in our study of SATS participants with mild AD [2]. Hallucinations and delusions are common in the more advanced stages of AD; thus, these symptoms and antipsychotic medication might not affect the outcomes in milder forms of the disease. Psychotic symptoms and the use of antipsychotics have shown a relationship with a faster rate of cognitive impairment and worse prognosis in earlier studies of patients with AD [29, 30], which supports our findings. Living alone implied an additional 3-point mean reduction of the outcome in ADAS-cog score in individuals with moderate AD in the present study, but this finding was not observed in the group with mild AD. However, participants with mild AD who lived alone exhibited more rapid worsening in IADL compared with those living with family [2]. In mild dementia, IADL deficits were more strongly associated with impairment in performance than in initiative [31]; thus, individuals with mild AD living alone could be dependent on support to maintain their performance of daily activities. Possible effects of solitary living, such as loneliness, social isolation and apathy, might also negatively affect disease progression. Apathy is more prevalent in the moderate/severe stages of dementia and has been reported as a predictor of faster cognitive decline [32]. This symptom might have a larger influence on the speed of cognitive impairment in the moderate stage, which might explain our findings. Furthermore, higher cognitive status in community-living elderly people is related to increased social support; that is, marital status and perceived positive support from friends [33]. We recently found that patients with mild AD living alone received an equal amount of home-help services as those with moderate AD [34]. These observations emphasize the risk of isolation and lack of mental stimulation among the vulnerable solitary-living care recipients with more advanced AD.

In this study of moderate AD, a higher mean dose of ChEI irrespective of the agent demonstrated a more favourable long-term outcome in both cognitive and functional capacities. Studies including participants in the mild and mild-to-moderate stages of AD from our group and others have previously reported this association [2, 7, 35, 36], which is considered essential knowledge for clinicians. Patients with more advanced AD have exhibited a better short-term response to ChEI therapy than those with milder disease in randomized trials and observational studies [4, 6]. A larger reversible cholinergic deficit in the more severe stages of AD could be a potential explanation [37]. The current longitudinal study shows the clinical importance of using optimal doses of ChEIs when treating individuals with moderate AD.

The strengths of the observational, prospective SATS are the large sample and well-structured semi-annual assessments of different aspects of AD progression over 3 years after the start of ChEI therapy. ‘Real-world’ AD outpatients with concomitant disorders and medications from 14 memory clinics across Sweden were enrolled. All participants were continuously treated with ChEIs during the study, and compliance in the SATS was high, which was investigated via an analysis of the level of the plasma concentration of the drug [14]. The Swedish health care system and its community-based services are publicly funded for all residents of Sweden [38], which assumes a representative selection of patients with AD and that the services used reflect the individuals’ actual needs for formal help, irrespective of socio-economic status. Like other longer-term naturalistic studies of AD, the limitations are that the SATS was not placebo controlled because of ethical concerns or was not randomized with respect to ChEI drug agent. Specialists in dementia disorders decided on the type of ChEI and dose for each participant, in agreement with the standards used in a routine clinical setting. Individuals who discontinued the study might have a worse prognosis than the completers, suggesting that the drop-outs had less benefit from ChEI treatment.

To our knowledge, no studies have previously compared the longitudinal cognitive and functional outcomes between patients with mild and moderate stages of AD; therefore, additional studies are warranted to confirm our findings. The potential effect of ChEI in various disease stages and possible risk factors that might alter the prognosis, such as co-morbidity and concomitant medications, need further investigation. This knowledge is essential to assess the effectiveness of and provide realistic expectations for new potentially disease-modifying AD therapies directed at different stages of AD. Baseline-dependent statistical models with the variance explained to a large extent were provided in the present study and could be used to predict the mean cognitive and functional outcomes for a ChEI-treated cohort with moderate AD in forthcoming studies of, for example, long-term combination therapy.

## Conclusions

A comparison of various aspects of disease progression between mild vs. moderate AD was reported in this observational 3-year study. The cognitive decline was significantly faster among the participants with moderate AD using the ADAS-cog scale, but not the MMSE test, indicating that the measure was dependent on the scales used. Deterioration on the IADL scale was similar between the different disease stages examined; however, the rate of basic ADL impairment was more pronounced in the group with moderate AD. This finding has important clinical implications, underlining the importance of functional assessments in the early stages of AD to avoid safety issues (e.g. driving and financial capacity) and to offer these impaired individuals the necessary formal care. Use of NSAIDs/acetylsalicylic acid was a protective factor for better cognitive outcome in moderate AD, suggesting that patients with greater neurodegeneration and cerebral inflammation have additional advantages of longer-term treatment with these drugs. Solitary living was a risk factor for more rapid cognitive progression in the moderate cohort, underlining the risk of apathy and social isolation among individuals with more advanced AD. A higher mean dose of ChEI was independently associated with slower cognitive and functional decline among the group of participants with moderate AD, stressing the importance of also optimizing the dose for patients in this group. Prediction models designed for the cohort with moderate AD are presented for the first time in this study; these models might be a useful tool with which to estimate the mean cognitive and functional outcomes that may be expected using ChEI monotherapy over extended times.

## Abbreviations

AD: Alzheimer’s disease
ADAS-cog: Alzheimer's Disease Assessment Scale—cognitive subscale
ADL: Activities of daily living
APOE: Apolipoprotein E
ChEI: Cholinesterase inhibitors
CI: Confidence interval
CIBIC: Clinician Interview-Based Impression of Change
IADL: Instrumental Activities of Daily Living Scale
MMSE: Mini-Mental State Examination
NINCDS-ADRDA: National Institute of Neurological and Communicative Disorders and Stroke–Alzheimer’s Disease and Related Disorders Association
NSAID: Non-steroidal anti-inflammatory drug
PSMS: Physical Self-Maintenance Scale
SATS: Swedish Alzheimer Treatment Study
